# Infants Need More Variety – Increased Data Acquisition with Reduced Participant Attrition in Infant ERP Studies

**DOI:** 10.3389/fpsyg.2013.00117

**Published:** 2013-03-18

**Authors:** Manuela Stets, Mike Burt, Vincent M. Reid

**Affiliations:** ^1^Developmental Cognitive Neuroscience Lab, Department of Psychological and Brain Sciences, Indiana UniversityBloomington, IN, USA; ^2^Department of Psychology, Durham UniversityDurham, UK; ^3^Department of Psychology, Lancaster UniversityLancaster, UK

**Keywords:** infant, ERPs, stimulus presentation, data-collection, attrition, methodology

## Abstract

Infant ERP studies often feature high attrition rates with large numbers of trials excluded from statistical analyses. The number of experimental conditions is conventionally limited to reduce the test-sessions’ durations and to ensure that reasonable trial-numbers will be obtained for each condition. Here, we designed an ERP study involving eight conditions originating from three previously published studies and presented them to 18 1-year-olds. We expected to replicate original results at least partly. Additionally, we were interested in the effect this novel method of stimulus presentation would have on infant attention. Due to the requirement for sustained attention, interest may decrease. Alternatively, the stimulus-variability may extend attention, allowing the acquisition of more valid trials. Our main finding was that the variability of the stimulus presentation sustained the infants’ attention beyond normal parameters. This is apparent from the markedly increased number of artifact-free trials obtained and from the substantially decreased attrition rates. Results from a gap-/no gap-task were fully replicated whereas others, related to face-processing, were replicated in part. Additionally, effects that were not reported in the original studies were found. This is most probably due to interference in the information processing between these conditions. The results show that presenting infants with varied stimuli extends their attention, allowing the acquisition of at least four times more data than via current infant ERP methods. However, stimuli from separate sub-experiments must be cognitively and perceptually distinct, otherwise contamination between related factors will occur.

## Introduction

According to general agreement among developmental psychologists working with infant populations, infants’ attention span is very limited and hard to sustain over an extended period of time (e.g., Thierry, 2005; DeBoer et al., 2007). Furthermore, it is frequently stated that it is easy to overload an infant’s cognitive system with too much information or with task-demands that are too complex for a specific age group (Nelson and Collins, 1991; Parise et al., 2008). Additionally, there seems to be common agreement that substantial attrition rates are to be expected when studies are conducted with infant populations (e.g., DeBoer et al., 2007; Picton and Taylor, 2007). Therefore, a common practice – particularly among researchers using ERP methodologies with infants – is to limit the number of experimental conditions and the duration of the individual stimuli that are presented to the participants. Moreover, these strategies are not only frequently used but also frequently recommended in methodological literature (Picton et al., 2000; DeBoer et al., 2007; Hoehl and Wahl, 2012).

One of the most frequent contributors to an infant ERP study’s attrition rate, as stated in published articles, is fussiness. However, as of yet, no comprehensive definition has been provided for this concept. Most likely, fussiness is involved in a marked decline in attention toward the presented stimuli. If an infant displays negative affect prior to observing a minimum number of trials, the study is terminated and the infant is marked as “fussy” for the purposes of the study. However, in their recent meta-analysis of 149 articles on infant ERP-studies, Stets et al. (2012) could not find any correlation between study-features such as the number of conditions or the stimulus duration and the attrition rate that was attributed to fussiness. This was true irrespective of the age group tested. The only obvious impact on attrition rate in the examined infant ERP studies was the nature of the stimuli (purely visual, purely auditory, or auditory and visual combined). The age of the published article itself was also a predictor of attrition rate, as was the number of participants that had been tested in total for a particular study or experimental group. Infants participating in a study using purely visual stimulation were twice as likely to have to be excluded from the study’s final analysis than those participating in a study using purely auditory stimulation. Also, the attrition rate tended to be lower if a study had been published recently and was higher if the total number of infants tested was high (see Stets et al., 2012).

In an attempt to investigate the impact of study design on infant attention, we created an ERP study with eight experimental conditions. Currently, the most frequently used strategy is to present infants with stimuli from two, typically very similar, experimental conditions which only differ in the experimental manipulation. However, as can be seen in Stets and Reid (2011), this repetitive presentation of very similar stimuli can eventually result in changes in attention-allocation toward the stimuli, which is likely due to habituation, thereby producing unforeseen changes in the ERP measures for each condition. We hypothesized that a more variable and, consequently, more engaging stimulus presentation would help to sustain infant attention over the course of a test-session. Therefore, we presented a group of 12- to 13-month-old infants with stimuli originating from three theoretically distinct research articles (Csibra et al., 2000; Halit et al., 2003; Hoehl et al., 2008). We selected this particular age range based on the assumption that younger infants would probably not tolerate such a multi-condition paradigm well and would cease to cooperate early in the test-session due to too high demands on the cognitive system (e.g., DeBoer et al., 2007). However, only two studies using purely visual stimuli have been published with this age group (i.e., Csibra et al., 2000; Halit et al., 2003). Moreover, due to lab-specific practical considerations (i.e., the test-sessions did not take place in a sound-proof cabin), we decided that we did not want to introduce an auditory component into the stimulus presentation. Therefore, as we did not know of any other infant ERP study using purely visual stimuli with 12-month-olds as well as an electrode setup which was comparable to ours (i.e., the replicability of channel-groups used in the respective analyses), and because the stimuli were readily available to us, we included the stimuli originating from Hoehl et al. (2008) even though they had not been tested with this age group before. As Csibra et al. (2000) and Halit et al. (2003) had tested groups of 12-month-olds, we aimed to replicate the results of these original articles at least in part. However, the main focus of the current study is the methodological issue of designing a paradigm that may potentially improve the ratio between the effort needed to conduct an infant ERP study and the amount of data that will result from it. In the following, the analyses will be presented in sequential order to make the methods and results used for the respective analyses more transparent for the reader.

## Methods – General

The stimuli depicting the eight conditions used in the present study originated from three theoretically unrelated research articles. Therefore, we decided to treat them as separate studies. In the following, we will first describe the general methods that were common to all of the conditions and then explain the specifics of the separate studies in more detail.

### Participants

We tested a total of 18 typically developing infants aged between 367 and 396 days (*M* = 381.61 days, SD = 9.66 days). All of the participants had been born full-term (between 37 and 42 weeks of gestation) and with a normal birth-weight (>2500 g). Twelve of the infants were male, six were female. Thirteen participants contributed data to all of the analyses. The attrition rates and exclusion criteria will be explained below in the respective sections for the individual sub-studies. Written consent for study participation had been provided by the parents or caregivers accompanying the infants prior to the test-session.

### Procedure

For the entire duration of the test-session, the infants were seated on their caregiver’s lap in a dimly lit, quiet testing-booth. The viewing distance to the 17′′ screen presenting the stimuli was 70 cm. The eight conditions were presented in an intermixed design. Two sets of eight stimuli were created with each set containing one stimulus per condition. The only differences between these two sets were the order of the conditions and the stimuli that represented the individual conditions. Individual stimuli depicting the two conditions originating from Csibra et al. (2000) and Hoehl et al. (2008), respectively, did not follow each other immediately. However, as the four conditions originating from Halit et al. (2003) made up 50% of the entire stimulus presentation, there were instances of two stimuli depicting one of these conditions following each other. There were no instances of three stimuli belonging to the same original study following each other.

An uninterrupted run through the entire stimulus presentation lasted for approximately 11 min with each condition being presented 34 times (total number of stimuli = 272). The duration of the inter-stimulus interval was 1000 ms in all cases. When an infant showed obvious signs of fussiness or boredom he or she was given a short break, and the test-session was discontinued when the infant’s attention to the screen could not be recaptured. To control for the infants’ attention to the screen, their behavior was video-recorded onto the hard-drive of a DVD-recorder for later off-line coding.

### EEG recording and analysis

The EEGs were recorded continuously from 32 scalp locations at a sampling rate of 1000 Hz using Ag-AgCl ring-electrodes, arranged according to the 10–20 system, and a conductive gel. During data-acquisition, the recordings were referenced to FCz. The data were amplified with a NeuroScan SynAmps amplifier, and vertical and horizontal electro-oculargrams were recorded bipolarly to control for artifacts resulting from eye-movements. The data were filtered with a band-pass filter with the upper edge set at 0.3 Hz and the lower edge set at 30 Hz. Baseline-correction was conducted using a baseline of 100 ms.

## Analysis 1 – The Spike-Potential (Csibra et al., 2000)

### Methods

#### Participants

Fourteen of 18 participants entered the final analysis. Four infants had to be excluded from the analysis because they did not reach the minimal criterion of providing at least 10 artifact-free trials to the averaging process due to fussiness or inattentiveness (*n* = 3) or because the averaged data contained frequency-artifacts (*n* = 1). (We define “fussiness” as the state when the infant shows signs of unhappiness with the test situation such as a negative facial expression, agitated movements, vocalizations, or trying to remove single electrodes or the entire cap.) The overall attrition rate for this analysis was 22.22%. The mean-number of artifact-free trials contributed by the infants was 24.21 (range: 16–39). These numbers refer to both of the conditions that contributed data to this analysis in conjunction as the statistical analysis was collapsed for the conditions as was the case in the original study (G. Csibra, personal communication, September 25, 2012).

#### Stimuli

The stimuli were created using Adobe Photoshop based on the descriptions provided in Csibra et al. (2000). One of three different objects (a red rubber-boat, a wooden rattle, or a yellow rubber-duck) appeared in an upright orientation in the center of the screen set on a gray background as had been practiced in Csibra et al. (2000). For a total duration of 990 ms, these objects were spun anti-/clockwise in the center of the screen while concurrently decreasing in size. In order to create a smooth spinning movement, we presented nine different static images of the same object, each turned by an angle of 90° and slightly reduced in size for 110 ms.

**Figure 1:**
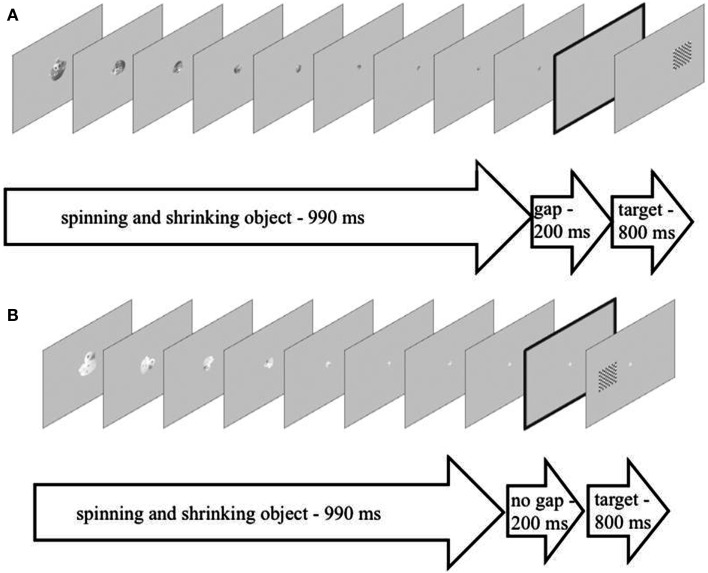
**(A)** An example of the setup of a trial depicting the experimental condition with a gap (presented for 200 ms) between the spinning and shrinking objects (presented for 990 ms, nine individual images presented for 110 ms each) and the target checkerboard (presented for 800 ms). **(B)** An example of the setup of a trial depicting the experimental condition without a gap (presented for 200 ms) between the spinning and shrinking objects (presented for 990 ms, nine individual images presented for 110 ms each) and the target checkerboard (presented for 800 ms).

For the gap-trials, the last image depicting the spinning object in an upright position and at its smallest size (approximately 2 cm × 2 cm) was presented for 110 ms and then disappeared leaving the screen blank except for the gray background-color. After 200 ms a black-and-white checkerboard of approximately 5 cm × 5 cm edge-length appeared on either the left or the right side of the screen for 800 ms with the center of the screen being empty (see Figure 1A). In the trials without the gap, the last image depicting the spinning object in an upright position and at its smallest size was presented for 110 ms to account for the last 110 ms of the 990 ms-spinning-motion and then for another 200 ms in order to bridge the gap. Again, after these 200 ms a checkerboard appeared to one side of the screen while the previously spinning object was still present in the center of the screen. This display was presented for 800 ms (see Figure 1B). The spinning-direction and the side on which the checkerboard appeared were counterbalanced across trials and conditions.

#### Data analysis

**Figure 2:**
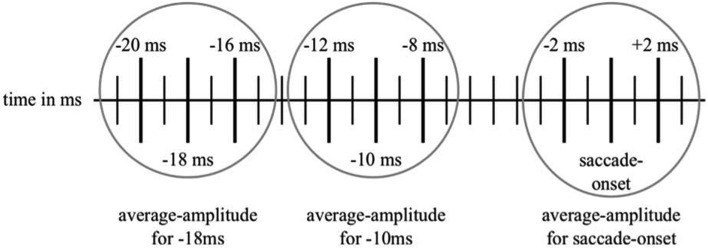
**An illustration of our sampling method used for collecting the data for the amplitude-comparison between saccade-onset and the time-points of −10 and −18 ms before saccade-onset**. The highlighted vertical lines represent the time-points from which the amplitudes have been noted. Then, these three amplitudes were used to calculate the mean-amplitudes for the respective time-points.

Following the methods outlined in Csibra et al. (2000), we re-referenced the data to the average-value of all the channels used. In order to identify the trials that would be usable for the statistical analysis, the video-recordings of each individual trial of the gap- or no gap-conditions were manually inspected frame by frame for participants’ potential saccades toward the checkerboard. The first frame counted was the one when either the gap or the bridged period started. Each frame had a duration of 40 ms. Therefore, if a shift in a participant’s eye-gaze direction was detected in frame 8, for instance, the onset of the saccade was noted as having a latency of 320 ms. According to this approximate latency-value of the saccade-onset, we identified the positive peak closest to this latency in each individual trial. Then, we took note of the exact latency of the respective saccade-onset and of the amplitude at this latency and, as the sampling rate in the original article had been set at 500 Hz rather than at the 1000 Hz that we used, at ±2 ms around this latency. The same procedure was followed for the amplitudes at 10 and 18 ms prior to the established saccade-onset. Therefore, we had three amplitude-values for each of the three latency-periods (see Figure 2 for an illustration of the sampling method). Finally, we calculated the average amplitude-values for these three time-points (i.e., saccade-onset, 10 and 18 ms before saccade-onset). Following Csibra et al. (2000), this analysis was only conducted for one channel, namely Pz. We discarded any trials in which the participants either did not look at the screen at the onset of the gap- or the bridged period or when they had seen it but did not shift their eye-gaze toward the checkerboard within 1000 ms or at all. Moreover, trials which were contaminated by movement- or other artifacts and trials when the participants shifted their eye-gaze direction to a point off-screen within the 1000 ms were excluded from the average as well.

In the analysis presented in Csibra et al. (2000), the amplitude-difference between the time-points of the saccade-onset and at −10 and −18 ms had been compared collapsed across conditions (i.e., treating the trials originating from the two conditions alike). This had been done as there were no differences between conditions except for the latencies of the spike-potential and the respective saccade. Saccades in the No-Gap-trials frequently occurred slightly later than the ones in the Gap-trials due to the so-called “sticky-eyes” phenomenon. Following the procedure of the original article, we collapsed the data from our gap- and bridge-trials in our analysis as well (G. Csibra, personal communication, September 25, 2012). Therefore, we conducted two paired-samples *t*-tests: one comparing the average amplitudes obtained from the latencies at saccade-onsets to those from the time-point 10 ms prior to the respective saccade-onset and another comparing the average amplitudes obtained from the latencies at saccade-onsets to those from the time-point 18 ms prior to the respective saccade-onset.

### Results

The cognitive component under investigation in Csibra et al. (2000) was the spike-potential – a positive peak located at 10 and 18 ms prior to a saccade-onset toward a target stimulus. As the spike-potential was present in both experimental conditions (i.e., trials with or without a gap), the authors had conducted their statistical analysis collapsed across these two conditions. Including the data from 10 out of 26 participants (attrition = 61.5%), they reported significant differences in amplitude between the latencies at which the saccade-onsets appeared compared to the latencies at 10 and at 18 ms before the respective saccade-onsets; *F*_−10 ms_(1, 9) = 22.5, *p* < 0.002 and *F*_−18 ms_(1, 9) = 8.1, *p* < 0.02 (Csibra et al., 2000, p. 1071). The data from 14 of our 18 1-year-olds replicated these results. The results of our amplitude-comparison between saccade-onsets and the latency at −10 ms to the respective saccade-onsets was *t*_−10 ms_(1, 13) = 5.19, *p* < 0.001. The results of our amplitude-comparison between saccade-onsets and the latency at −18 ms to the respective saccade-onsets was *t*_−18 ms_(1, 13) = 4.04, *p* = 0.001 (see Table 1 for a list of the respective amplitudes from the individual participants).

**Table 1:** **An overview over the mean-amplitudes established from the 14 1-year-olds who contributed data to our analysis of the spike-potential in comparison to the time-points at −10 and at −18 ms before saccade-onset**.

Participant-number	Mean-amplitude at saccade-onset in μV	Mean-amplitude at −10 ms in μV	Mean-amplitude at −18 ms in μV
02	25.235	58.421	32.603
03	4.859	11.582	6.813
04	−7.227	2.329	−6.065
06	−6.038	−1.623	−5.382
07	18.631	30.84	22.056
08	1.694	13.989	4.072
09	−6.362	17.526	−4.409
11	13.566	20.53	16.684
12	−9.325	18.024	−7.336
13	3.298	33.310	5.63
14	−2.818	7.101	−0.57
16	−4.802	23.715	0.673
17	−31.574	47.115	−24.43
18	5.634	14.931	6.454

### Discussion

Csibra et al. (2000) showed that the spike-potential could be reliably measured at 10 and 18 ms before a saccade-onset toward a target stimulus in both a group of adults and in a group of 12-month-olds. The results from our group of 12-month-old infants replicated Csibra et al.’s (2000) results. There were significant differences in amplitude between the time-points at saccade-onsets and at 10 and 18 ms prior to the respective saccade-onsets. Moreover, we could also replicate the so-called “sticky-eyes” phenomenon, which means that the infants took longer to shift their eye-gaze from the center of the screen toward the checkerboard on the side when the gap was bridged. Therefore, the presence of the other six experimental conditions did not impact on the way the gap- or bridged trials were processed by our group of 12- to 13-month-olds.

## Analysis 2 – The N290 and the P400 (Halit et al., 2003)

### Methods

#### Participants

Fifteen of 18 tested participants entered the final analysis. Three infants had to be excluded from the analysis because they did not reach the minimal criterion of providing at least 10 artifact-free trials to the averaging process due to fussiness or inattentiveness (*n* = 3). Therefore, the overall attrition rate for this analysis was 16.67%. The mean-number of artifact-free trials contributed by the infants across conditions was 13.89 (range: 10–25). For individual conditions the mean-numbers of trials contributed was as follows: *M*_HumanUpright_ = 14.4, SD = 3.94; *M*_HumanInverted_ = 13.27, SD = 3.13; *M*_MonkeyUpright_ = 14.4, SD = 4.09; and *M*_MonkeyInverted_ = 13.47, SD = 2.72.

#### Stimuli

The raw stimuli used in the original study were provided to the authors by Olivier Pascalis. We edited these using Adobe Photoshop according to the descriptions given in Halit et al. (2003). We presented faces of humans (*n* = 3) and of macaque-monkeys (*n* = 3). Each of these faces appeared in an upright or in an inverted orientation in the center of the screen for 1000 ms. In accordance with Halit et al. (2003), the background-color was set to gray. The faces had a neutral expression and their gaze was directed straight ahead toward the infants. The images showed the faces from a fully frontal view.

#### Data analysis

Following the procedure described in Halit et al. (2003), we re-referenced the data to the average-value of all the channels used. The data were inspected manually for artifacts, and trials in which a participant had not been attending to the screen or displayed movement- or eye-artifacts were rejected. For the original article, the statistical analysis had been conducted on the mean-amplitudes from six topographically distinct channel-groups. As our EEG-system did not feature the same number of electrodes as the one used in the original study, we decided to constrain our analyses to one channel-group per hemisphere that would approximately cover the same posterior areas as the original article, based on 10–20 locations. Therefore, we compared the data from one left and one right medial channel-group featuring the following electrodes: P7, P3, and O1 (left medial) and P8, P4, and O2 (right medial), respectively.

Congruent with Halit et al. (2003), we analyzed the data originating from these four conditions for the N290 and for the P400. For the N290-analysis, we extracted the most negative peak for each of the above-mentioned electrodes in the time-window from 140 to 380 ms after stimulus-onset. Then, we calculated one mean per channel-group from the negative peaks that had been obtained for the three electrodes in each channel-group. This mean-amplitude entered the statistical analysis for the N290. Moreover, we took note of the latencies at which the negative peaks had occurred at the individual channels and entered the latency of the most negative amplitude in the analysis for each channel-group. For the analysis of the P400, we determined the most positive peak for each of the above-mentioned electrodes for the time-period from 380 to 584 ms after stimulus-onset. Then, we calculated one mean for each of the two channel-groups from the positive peaks that had been obtained for each of the three electrodes in the channel-groups. These two means then entered the statistical analysis. The analysis of the P400-latency was prepared as described for the N290 latency except that the latency for the most positive amplitude was selected. For both components, the N290 and the P400, the analysis of the potential difference in amplitude was conducted using a 2 × 2 × 2-repeated measures ANOVA with the factors Species (Human and Monkey), Orientation (Upright and Inverted), and Location (Left medial and Right medial). The same statistical test was used to establish potential differences in the latencies of the N290 and of the P400.

### Results

#### The N290

**Figure 3:**
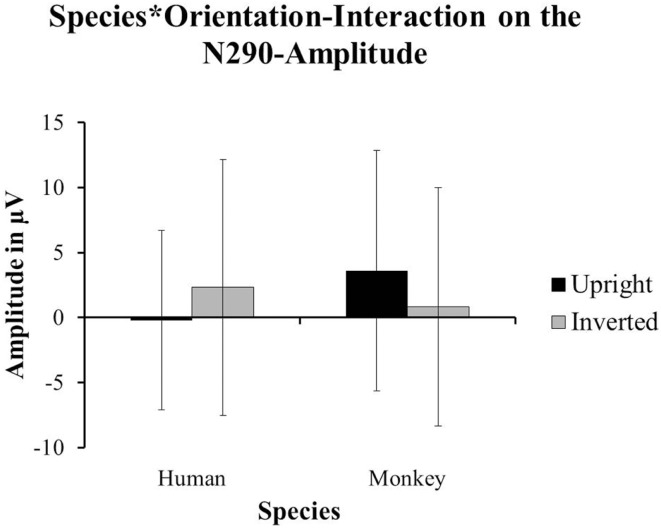
**The data from our group of 1-year-olds showed a tendency towards an interaction of Species and Orientation on the amplitude of the N290 [*F*(1, 14) = 3.36, *p* = 0.088]**. The upright human faces (±SD) yielded more negative responses than the inverted human faces (±SD). However, the inverted monkey-faces (±SD) yielded the more negative responses compared to the upright monkey-faces (±SD).

The authors of the original paper analyzed the differences in amplitude for two components: the N290 (between 140 and 380 ms after stimulus-onset) and the P400 (between 380 and 584 ms after stimulus-onset). Using the data from 26 out of 85 participants (attrition = 69.4%), they reported a main-effect of Species on the amplitude of the N290 [*F*(1, 24) = 15.57, *p* < 0.01; cf. Halit et al., 2003, p. 1184] with the responses to human faces being significantly more negative than those for monkey-faces. Moreover, they found the Orientation of the human and monkey-faces to have an impact on the amplitude of the N290 as well. A *post hoc* test revealed that inverted faces yielded more negative responses than upright stimuli; *F*(1, 24) = 7.21, *p* < 0.02; (p. 1185). The data from 15 of our 18 participants showed a marginal tendency toward an interaction between Species and Orientation impacting on the amplitude of the N290 [*F*(1, 14) = 3.36, *p* = 0.088]. For human faces, the amplitude of the N290 was more negative for upright faces (*M* = −0.226 μV, SD = 6.906) compared to the inverted ones (*M* = 2.32 μV, SD = 9.833). For monkey-faces, the amplitude of the N290 was more negative for the inverted faces (*M* = 0.832 μV, SD = 9.175) compared to the upright ones (*M* = 3.599 μV, SD = 9.268; see Figure 3).

With respect to the latency of the N290, Halit et al. (2003) reported a main-effect of Species [*F*(1, 24) = 16.00, *p* < 0.005; p. 1185] with the latencies for human faces being significantly longer than those for monkey-faces. The Orientation of the faces did not have an effect on the latency of the N290 in the original study. The data from our group of 12- to 13-month-olds did not show any differences in latency for the N290.

#### The P400

**Figure 4:**
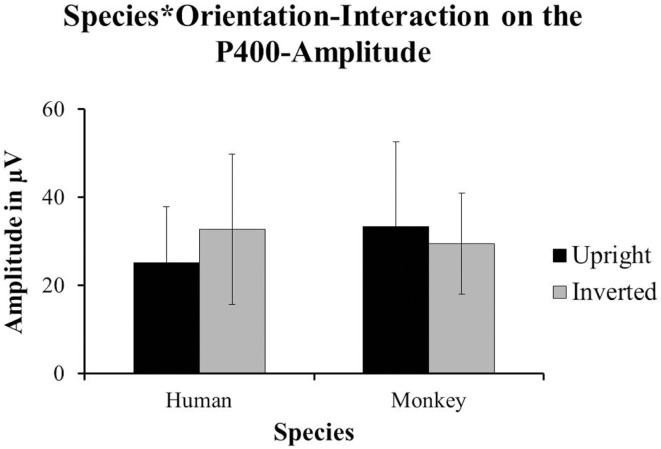
**The data from our group of 1-year-olds showed a significant interaction between Species and Orientation impacting on the amplitude of the P400 [*F*(1, 14) = 5.39, *p* = 0.036]**. The inverted human faces (±SD) yielded the more positive responses than the upright human faces (±SD). However, the upright monkey-faces (±SD) yielded the more positive responses compared to the inverted monkey-faces (±SD).

Halit et al. (2003) did not find any effects on the amplitude of the P400 in their data. Our data revealed a strong significant interaction between Species and Orientation that impacted on the amplitude of the P400; *F*(1, 14) = 5.393, *p* = 0.036. In our data, the P400-amplitudes were significantly more positive for inverted human faces (*M* = 32.705 μV, SD = 17.083) compared to upright human faces (*M* = 25.111 μV, SD = 12.749), whereas upright monkey-faces (*M* = 33.327 μV, SD = 19.316) yielded significantly more positive responses than inverted monkey-faces (*M* = 29.426 μV, SD = 11.474; see Figure 4).

**Figure 5:**
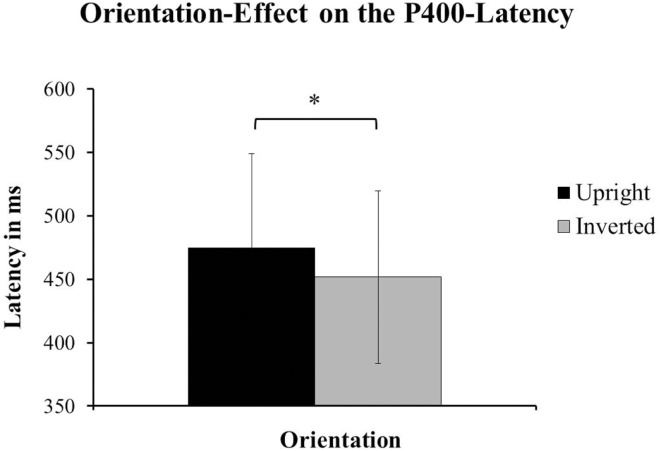
**The data from our group of 1-year-olds showed a significant effect of Orientation on the latency of the P400**. The faces presented in an upright orientation (±SD) had significantly longer latencies for the P400 than the inverted faces [±SD; *F*(1, 14) = 6.22, *p* = 0.026]. This effect was found irrespective of species.

**Figure 6:**
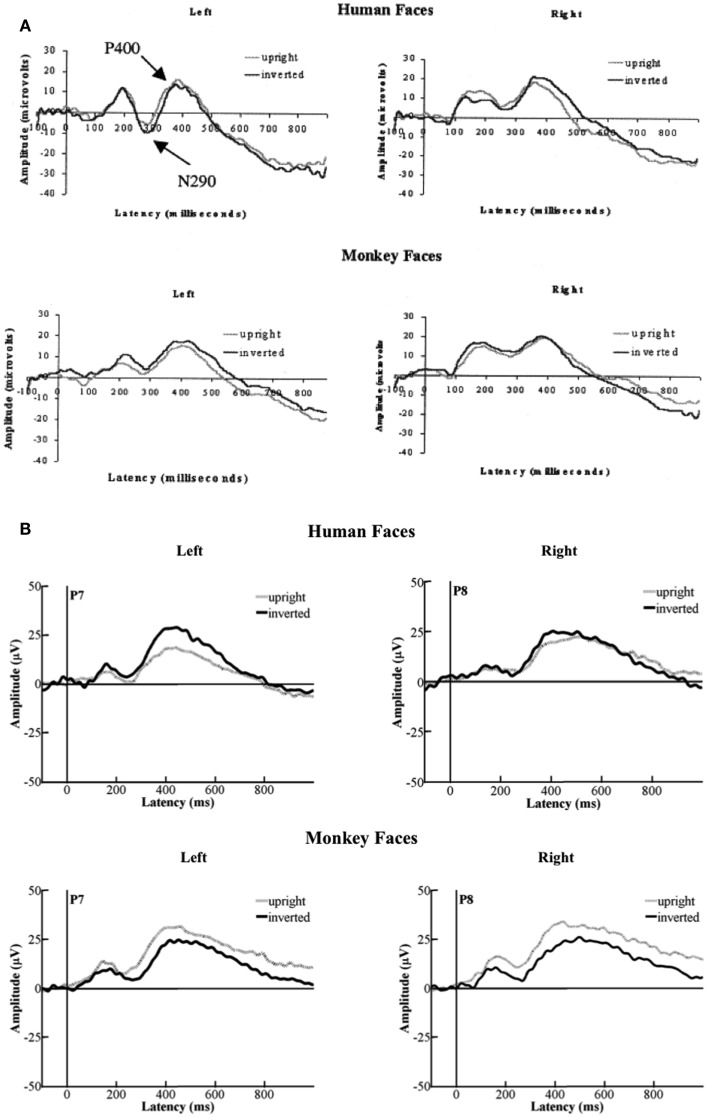
**(A)** Depicts the grand-average ERPs that Halit et al., 2003, p. 1183; Figure 1) had deducted from their data. **(B)** Illustrates the grand-average ERPs that we deducted from the data from our group of 12- to 13-month-olds. As can be seen, the morphologies of the original ERPs and ours are very similar. However, the directionalities in terms of which condition elicited the more negative or positive responses are reversed.

With respect to the latency of the P400, the authors of the original paper reported a main-effect of Species [*F*(1, 24) = 11.28, *p* < 0.01; Halit et al., 2003, p. 1185] with the P400 for human faces having a shorter latency than the one for monkey-faces. Our data showed a significant effect of Orientation on the latency of the P400. Upright faces (*M* = 474.75 ms, SD = 74.164) had a significantly later P400 than inverted faced (*M* = 451.63 ms, SD = 67.999) irrespective of species [*F*(1, 14) = 6.221, *p* = 0.026; see Figure 5]. For a comparison of the ERPs reported in Halit et al. (2003) and those resulting from our data see Figures 6A,B below.

### Discussion

Halit et al. (2003) reported significant main-effects of species and orientation on the amplitude of the N290. They reported more negative amplitudes for human faces relative to the monkeys and more negative responses for inverted human faces compared to upright human faces. Moreover, a main-effect of species on the latency of the N290 was found. The data from our group of 1-year-olds did not show separate main-effects of species and orientation on either the amplitude or the latency of the N290. However, we found a tendency toward an interaction between these factors that was impacting on the amplitude of the N290 only. Additionally, while Halit et al. (2003) could not find significant differences in N290-amplitude for upright and inverted monkey-faces, our data showed a tendency toward a difference in N290-amplitude which was the opposite to our results for the human faces. The latency of the N290 remained unaffected by these four experimental conditions in our study. With respect to the P400, our results also differed from those presented in Halit et al. (2003). While the authors of the original paper did not report differences in the amplitudes of the P400, our data revealed a significant interaction of species and orientation for the P400-amplitude. Moreover, contrary to the main-effect of species on the P400-latency reported in the original paper, we found a main-effect of orientation, not species, on the latency of the P400.

As can be seen in Figure 6B, despite the differences in the effects found, the ERPs sourced from our data are morphologically similar to those presented in Halit et al. (2003). Therefore, in terms of the morphology of the resulting waveform, our overall results are broadly in alignment with those reported in the original article. However, the fact that the number of conditions in our study was greater than the original study might have contributed to the discrepancies between the results of the original study and our own. It is possible that the cognitive processes involved in the processing of the other experimental conditions may have interfered with the processing of the upright and inverted human and monkey-faces, and vice versa. This may be the case given that other stimuli also involved faces (i.e., the Toward- and Away-conditions). This may have had an impact on the way the human and monkey-faces were processed by our group of participants – even though the social contexts depicted in the stimuli are very different from one another. Alternatively, given the potential changes in ERP components as shown in Stets and Reid (2011) or Nikkel and Karrer (1994), the differences in the effects and interactions found may be partly due to the minimum criterion applied in the original study and here. The infants who had been included in the analysis in Halit et al. (2003) had to contribute at least 25 artifact-free trials for each of the four conditions. Here, due to the naturally lower number of trials that could be presented per condition, the minimum criterion was set to 10. Consequently, it could be argued that the separate main-effects reported in Halit et al. (2003) were derived from interactions between the factors of species and orientation in the earlier stages of the experimental session. This argumentation may also be supported by the similarity between the waveform morphologies that were derived from our data and those presented in the respective original studies (see Figure 6 above).

## Analysis 3 – The Nc and the PSW (Hoehl et al., 2008)

### Methods

#### Participants

Fourteen of 18 tested participants entered the final analysis. Three infants had to be excluded from the analysis because they did not reach the minimal criterion of providing at least 10 artifact-free trials to the averaging process due to fussiness or inattentiveness (*n* = 3). One infant was excluded due to excessive frequency-artifacts in the data (*n* = 1). Therefore, the overall attrition rate for this analysis was 22.22%. The mean-number of artifact-free trials contributed by the infants across these two conditions was 13.22 (range: 10–22). Trial-contribution for the two individual conditions was as follows: *M*_Toward_ = 12.93, SD = 3.52; and *M*_Away_ = 13.5, SD = 3.7.

#### Stimuli

We were provided with the images that had been used in the original study by Stefanie Hoehl. The images depicting a female adult who is looking toward or away from a toy positioned at eye-level to the side of her head were presented for 1000 ms each. Additionally, we presented the same object that Hoehl et al. (2008) had used as a central attractor for 500 ms prior to the target stimulus. In accordance with Hoehl et al. (2008), the background-color was set to white both for the central attractor and for the target-stimuli.

#### Data analysis

Hoehl et al. (2008) analyzed the data of their 4-month-olds for potential differences in the peak-amplitudes and -latencies of the Negative component (Nc) between 400 and 600 ms after the onset of the target images. Moreover, they compared the mean-amplitudes of the time-window between 700 and 1000 ms after target-onset for a difference between the conditions in the Positive Slow-Wave (PSW).

After visual inspection of the individual averages in the obtained data for these two conditions, it was apparent that the peaks of the Nc frequently appeared slightly earlier than 400 ms after stimulus-onset. This can be explained with the developmental changes that are expected to have taken place between the ages of 4 months and 1 year of age (cf., Tomasello, 1995; Rochat and Striano, 1999; Taylor and Baldeweg, 2002). Therefore, we decided to shift the time-window for the analyses of the Nc-peak and its latency from 400 to 600 ms after stimulus-onset to the time-window from 350 to 500 ms after stimulus-onset. Moreover, due to a slight difference in the EEG-equipment used, our left-frontal channel-group featured F3, FC5 (instead of FC3), and C3, and our right-frontal channel-group featured F4, FC6 (instead of FC4), and C4, respectively. The fronto-central channel-group consisted of Fz and Cz.

### Results

#### The negative component

The data of 17 of 64 4-month-olds tested in the original study (attrition = 73.4%) revealed a main-effect of Condition on the peak of the Nc for the time-window from 400 to 600 ms after stimulus-onset. The stimuli depicting the condition with the averted gaze showed significantly more negative responses relative to the Toward-condition [*F*(1, 16) = 4.8, *p* = 0.044; cf. Hoehl et al., 2008, p. 13]. Moreover, in the condition in which the adult was looking toward the toy, the Nc-peak had a significantly shorter latency than in the Away-condition [*F*(1, 16) = 5.52, *p* = 0.034; p. 13]. The 2 × 3-repeated measures ANOVAs on the data from 14 of 18 participants in our study did not show any effects of Condition on either the amplitude of the Nc-peak or on its latency for the time-window from 350 to 500 ms.

#### The positive slow-wave

**Figure 7:**
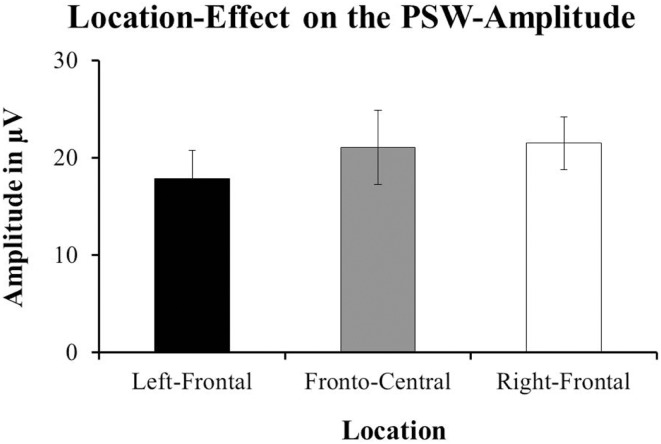
**The data from our group of 1-year-olds showed a tendency toward an effect of Location on the amplitude of the Positive slow-wave [PSW; *F*(1, 13) = 3.11, *p* = 0.061]**. The right-frontal channel-group (F4, FC6, and C4; ±SE) had more positive amplitudes compared to the fronto-central channel-group (Fz and Cz; ±SE) and the left-frontal channel-group (F3, FC5, and C3; ±SE).

**Figure 8:**
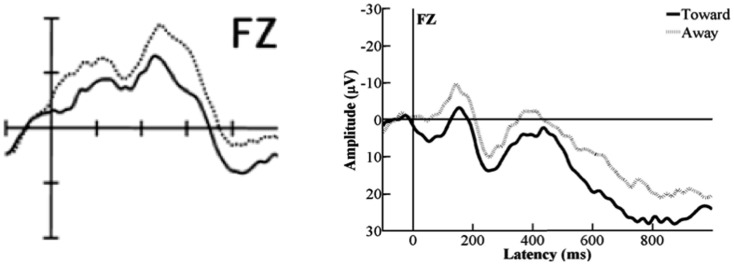
**A comparison between the ERPs deducted from the data published in Hoehl et al., 2008, p. 13; Figure 2; sample electrode depicted on the left) and those deducted from our data (sample electrode depicted on the right)**. As can be seen, the morphologies of the original ERPs and those from the present study are approximately similar.

Hoehl et al. (2008) reported a main-effect of Condition on the mean-amplitude of the PSW for the time-window from 700 to 1000 ms after stimulus-onset [*F*(1, 16) = 4.68, *p* = 0.046; cf. Hoehl et al., 2008, p. 12]. The Toward-condition showed a significantly more positive response than the Away-condition. Our data did not show an effect of Condition on the amplitude of the PSW. However, we found a tendency toward an effect of Location; *F*(1, 13) = 3.11, *p* = 0.061. The right-frontal channels (*M* = 21.467 μV, SE = 2.7) had more positive responses compared to the left-frontal (*M* = 17.868 μV, SE = 2.905) and fronto-central channels (*M* = 21.054 μV, SE = 3.841; see Figure 7). For a comparison of the ERPs reported in Hoehl et al. (2008) and those resulting from our data see Figure 8 below.

### Discussion

Hoehl et al. (2008) reported their group of 4-month-old infants to show significantly more negative responses toward the stimuli depicting the Away-condition. Following suggestions from earlier studies, this was interpreted as indicating that the infants had allocated more attention to these stimuli. However, even though more attention had been paid to the Away-condition, the processing of the Toward-stimuli was faster as shown in the significantly shorter latencies of the Nc for this condition. The results from our group of 1-year-olds did not show any differences in the amplitude or in the latency of the Nc. This may well be due to the age of our participants compared to the ones reported in Hoehl et al. (2008) and the developmental changes in terms of social information processing that will have taken place during these 8 months (e.g., Rochat and Striano, 1999).

Stets and Reid (2011) argued that the evolution of the morphology during the testing-session in the Nc in Hoehl et al.’s (2008) data might be due to the infants’ familiarity with the situation in which an adult is looking toward or away from an object. The authors suggested that the more negative responses would have been found for the Toward-condition in the early stages of an experimental session because this would be the social situation with which the infants would be more familiar. Someone looking away from a toy, however, is likely to be a situation that the infants would not have encountered during the first four postnatal months. Stets and Reid (2011) further argued that the shift in the directionality of the Nc-responses as a function of the time-course of the experiment might therefore be explained with the infants’ loss of interest in, or habituation to, the Toward-stimuli. Consequently, the infant would then allocate more attention to the Away-stimuli as this condition became more interesting relative to the Toward-condition. Following this logic, it is possible to conjecture that we did not find differences in the way the Toward- and Away-conditions have been processed by our group of 12-month-olds because, by this age, the infants would have had multiple experiences of both these social situations. Consequently, they could be expected to allocate equal amounts of attention to both these conditions.

Finally, Hoehl et al. (2008) found an effect of condition on the amplitude of the PSW. They interpreted this as an indication that the memory-trace for the toys, which had been highlighted by the adult’s gaze toward them, was relatively more imprinted than the trace for the objects that were not being looked at. We did not find an effect of condition on the amplitude of the PSW. However, we found a tendency toward an effect of location that was modulating the mean-amplitude of the PSW. The right-frontal channels showed the more positive responses compared to the other two channel-groups. Comparing this effect with the morphologies of the PSWs presented in Figure 2 in Hoehl et al.’s (2008, p. 13), one might argue that already at the age of 4 months a tendency toward this effect has been present. The ERPs of the right-frontal channels (presented on the right side of their Figure 2) appeared to be more positive compared to the ERPs for the fronto-central channel-group and reliably more positive than those for the left-frontal channel-group. However, apparently the difference was not a significant one in the group of 4-month-olds, with the current data suggesting that eight additional months would produce location effects for this task.

As was also the case for the experimental conditions originating from Halit et al. (2003), we found different results compared to the original article for the Toward- and Away-conditions (Hoehl et al., 2008). However, parallel to what we could show for the upright and inverted human and monkey-faces, the ERPs sourced from our set of data were morphologically similar to the ones presented in Hoehl et al.’s (2008; for a comparison see Figure 8). This indicates that our results are generally representative of the cognitive processes involved in processing such stimuli at an age of 12 to 13 months.

## Discussion – General

The main objective of the current study was to investigate the consequences of presenting a group of 1-year-old infants with eight highly variable experimental conditions rather than the normative two which are typically very similar to each other in every way apart from the experimental manipulation. According to common agreement among researchers (e.g., Thierry, 2005; DeBoer et al., 2007), infants should not be capable of meeting the high task-demands required due to the extended period of time needed for providing a suitable number of trials from viewing eight conditions. It would be expected that the infants’ interest in the stimulus presentation would decrease soon after the start of the experimental session. Consequently, the infants would cease to cooperate prior to the acquisition of data in the volume required for standard ERP analysis with this population (i.e., typically minimally 10 artifact-free trials per condition). Moreover, were sufficient trial-numbers to be acquired, the attrition rate experienced with such a paradigm would be expected to be substantial. Especially so as studies with the more typical two conditions frequently feature attrition rates between 50 and 75% (see Stets et al., 2012). Typical examples are the studies by Kobiella et al. (2008; age group = 7 months, number of conditions = 2, attrition rate = 55.3%), de Haan and Nelson (1997; age group = 6 months, number of conditions = 2, mean-attrition rate across all four experimental groups = 63.58%), and Jeschonek et al. (2010; age group = 8 months, number of conditions = 2, attrition rate = 74.6%).

In their meta-analysis of ERP studies with infants published between 1978 and 2010, Stets et al. (2012) reported a mean-attrition rate for infant studies of 49.2% (range: 0–83.8%, see their Table 2, p. 234) for the 181 experimental groups that had been included in their final analysis. This means that, when researchers plan to conduct an ERP study with an infant population, 50% of the time and resources allocated to data-collection will usually be spent on test-sessions from which no data will be obtained. This is a considerable amount of resources that could be spent on other studies and could, therefore, be used more effectively. However, as the attrition rates reported for our analyses clearly indicate, it is possible to make the process of collecting data from infants much more efficient. We were essentially conducting three studies simultaneously. Rather than the previously predicted effect of inducing fussiness in the participants due to the increased volume of presented stimuli, in actuality, presenting the infants with eight different experimental conditions appeared to help sustain the infants’ attention span. Therefore, as opposed to increasing the risk for attrition and for a premature end of the experimental session, the variability of the stimulus presentation increased the infants’ compliance and their ability to attend to the stimuli for a relatively longer period of time. Moreover, not only was it possible to test the infants for approximately 10 to 11 min, we were also able to extract at least 10 artifact-free trials for all of the analyses from the data of the majority of the infants.

This is also in line with Stets et al. (2012) finding that there were no correlations in infant ERP studies between the number of experimental conditions presented to the participants and either (1) the overall attrition rate reported for a study or (2) the nature of the stimuli or (3) the attrition rate due to fussiness. However, what the authors did report, amongst other effects, was the potential presence of a selection bias (see their Figure 2, p. 235). They found that the number of studies that have a larger SE (i.e., more variable data from the included participants of a smaller group of originally tested infants) and are published is much smaller than the number of published studies with less variation in the data (i.e., less variable data from few included participants of a large number of originally tested infants). This likely reflects a potential bias with respect to inclusion criteria and what kind of characteristics, such as a positive temperament, an infant must have in order to be included into a study’s data set. As has been mentioned in Stets et al. (2012), this draws the generalizability of the results into question.

As has been mentioned previously, the total number of stimuli presented in the current study when the stimulus presentation ran uninterruptedly was 272. A minimum of 70 artifact-free trials (25.7%) were obtained during the 11-min test-session for the infants who were included in our analyses. In infant EEG research, generally, the mean-number of artifact-free trials included in analyses is around 65 per condition (see Table 2 in Stets et al., 2012). However, there was great variation (SD = 182.19) between studies in the number of trials used per condition. Part of this variation is likely due to the 19 studies which used odd-ball paradigms (e.g., He et al., 2007) in which the often auditory stimuli were of short duration and were very well attended (*M* = 290.43, SD = 403.76, range: 8–1186). However, the mean-number of artifact-free trials included in the other 105 experimental groups reported in Stets et al. (2012) was much lower, 24.7 (SD = 10.8, range: 9–79.8) and is in line with the amount of data obtained in the current study.

Another important ratio is the percentage of usable trials relative to the number of trials presented to the participants per condition. As for the number of included trials, our ratio here (41.73%) is comparable to those reported in other studies using similar standard paradigms. Stets et al. (2012) reported that the mean-number of stimuli presented to a participant per condition was 117.8 (SD = 202.39) for 131 of the 181 experimental groups. As for the number of included trials, the SD is very high due to the groups originating from odd-ball paradigms^1^. Therefore, when calculating the percentage for how many of the mean-number of trials that have been presented per condition typically enter the final analysis on average, the result is 55.52%. However, calculating the same value based on the data from only the 70 experimental groups which did not originate from a study using an odd-ball paradigm and provided data on both the number of presented and included trials per condition, the value decreases to 44.18% per condition (*M*_presented_ = 53.51, SD = 35.21, range: 1–108; *M*_included_ = 23.64, SD = 9.36, range: 9–53). For the 18 experimental groups with odd-ball paradigms that provided data on both number of presented and included trials, the percentage of number of trials included on average from the number of trials presented on average is 73.17% (*M*_presented_ = 400.98, SD = 352.28, range: 1–800; *M*_included_ = 293.38, SD = 415.25, range: 8–1186). In the present study, the mean-number of trials presented per condition and analysis was 36.35 (SD = 4.06, range: 28.86–42) and the mean-number of trials included per condition and analysis was 15.17 (SD = 4.03, range: 10.86–24.29). This results in a percentage of usable trials per condition and analysis of 41.73% – again, a value that is comparable to the 44.18% from the 70 non-odd-ball experimental groups from Stets et al. (2012) meta-analysis. Therefore, despite presenting a larger array of experimental conditions, we were able to acquire the same amount of data per condition as seen in two-condition paradigms. Moreover, as we had eight conditions, the method used in the present study is four times more efficient for data acquisition than when contrasted with standard paradigms.

### Implications

Scientific work with infant populations requires large amounts of resources, due, in part, to often high attrition rates. Attrition is frequently caused by limited infant attention capacities, coupled with the requirements for minimum trial-contributions to individual ERP averages. Here, the variation in stimuli appears to decrease attrition rates. In our analyses, we experienced attrition rates that were between 25 and 30% lower than the average attrition rate typically reported (Stets et al., 2012). Furthermore, we demonstrated that it is possible to increase the amount of usable ERP data obtained from infant participants. Our results show that by presenting 12- to 13-month-olds with eight as opposed to the more typical two experimental conditions, the amount of artifact-free data to be obtained can be increased three to four times. We attribute this to the variability of the stimulus presentation, allowing the infants’ attention to be sustained for an extended period in comparison to previous studies. The approach outlined in the present study can therefore facilitate data acquisition in an infant population when contrasted to current approaches.

Another positive aspect of such a decrease in attrition rates is that a study’s results can potentially be seen as being more representative of the population. For example, Marshall et al. (2009) conducted an ERP study with three groups of 9-month-olds who had been rated on their temperament by their parents prior to testing. Apart from their finding that there were processing differences between the three groups, as a by-product, the authors noted that the attrition rates experienced for these groups differed substantially as well. The 9-month-old infants who had received negative ratings were much more likely to have to be excluded from the study’s analysis compared to those rated positively and to a control group which had not been rated prior to the test-session. Based on these data and on the likely selection bias reported in Stets et al. (2012), it seems apparent that representativeness of a study’s results cannot be assumed in all cases – especially, when the attrition rate is comparatively high. Consequently, using a paradigm that helps to decrease the likelihood for attrition can be instrumental in ensuring the generalizability of a study’s results.

With respect to replicating the results of the three original research studies, our data show varying results. While some of the effects were replicated (Csibra et al., 2000), others were not [e.g., our significant interaction between Species and Orientation affecting the P400-amplitude for upright and inverted human and monkey-faces where Halit et al. (2003) had not found any effects]. Particularly in the case of the conditions originating from Hoehl et al. (2008), the difference to the original findings might be due to the developmental changes between the ages of 4 and 12 months. An additional factor that might potentially have caused differences in results was interference between the face stimuli used in different conditions derived from different original experiments: both Halit et al. (2003) and Hoehl et al. (2008) included facial stimuli. Therefore, we would recommend great care in the selection of experimental conditions should such a paradigm as outlined in this article be used. The conditions presented to participants should cover distinct perceptual or cognitive domains in order to ensure that the processing of one set of conditions will not interfere with the processing of another set of conditions. For instance, it might be possible to mix two conditions on color-perception with stimuli involving social cognition, and a task on saccades. Furthermore, the use of purely auditory or multimodal stimuli intermixed with purely visual stimuli is possible. An alternative approach would be to present the participants with a small amount of experimental conditions but a large variety of different stimuli for each of the conditions. However, the variability of the stimuli would still be relatively limited as the stimuli could only be different to each other to a certain degree. Too much variability between the stimuli could potentially lead to unwanted differences in the ERPs toward those stimuli and, ultimately, in the responses toward the experimental conditions. Moreover, this approach had been implemented in the study reported in Jeschonek et al. (2010) and their attrition rate still reached a level close to 75%. Therefore, we would recommend caution with such an approach.

## Conclusion

In the present infant ERP study, we tested a group of 12- to 13-month-olds with eight rather than the more typical two experimental conditions in order to investigate the effects of applying such a paradigm on the infants’ capacity to contribute data during such a test-session. The conditions originated from three distinct research articles addressing theoretically unrelated aspects of cognitive development. As a consequence of the high variability in the stimulus presentation, we were able to collect a sufficient number of artifact-free trials to conduct statistical analyses comparable to each of the original analyses. Moreover, for each of these three analyses, we had improved attrition rates when contrasted with studies utilizing two conditions. The effects and interactions that we could draw from our data did not entirely match those reported in the original articles. However, the morphologies of our waveforms did match the original publications. We conjecture that discrepancies in the reported effects and interactions were a consequence of cross-experiment interference between those conditions that included facial and, therefore, social stimuli. However, whilst advising researchers to be cautious in their choice of experimental conditions, our results suggest that the improvements in data acquisition from presenting more varied stimuli for multiple conditions, coupled with the reduced attrition rates, outweigh the drawbacks that are inherent in presenting two conditions to participants. We are aware that our methodological results are preliminary and require refinement and validation through further studies also from other laboratories. Therefore, we would encourage the research community to apply similar paradigms with varying components (e.g., intermixing auditory and visual stimuli) in order to validate the approach that has been taken in this article.

## Conflict of Interest Statement

The authors declare that the research was conducted in the absence of any commercial or financial relationships that could be construed as a potential conflict of interest.

## Footnotes

^1^It needs to be noted that Stets et al. (2012) only presented their analysis collapsed for all types of experimental tasks and did not differentiate between experimental groups originating from standard and odd-ball paradigms. Therefore, the above numbers for the respective variables and types of experimental tasks were calculated for the present study.
